# Implementation of SiN thin film in fiber-optic sensor working in telecommunication range of wavelengths

**DOI:** 10.1038/s41598-021-00195-9

**Published:** 2021-11-17

**Authors:** Sandra Pawłowska, Jakub Gierowski, Bartłomiej Stonio, Marcin Juchniewicz, Mateusz Ficek, Michał Kruczkowski, Małgorzata Szczerska

**Affiliations:** 1 Department of Metrology and Optoelectronics, Faculty of Electronics, Telecommunications and Informatics, Gdansk University of Technology, 11/12 Narutowicza Street, 80-233 Gdansk, Poland; 2 Centre for Advanced Materials and Technologies CEZAMAT, Warsaw University of Technology, 19 Poleczki Street, 02-822 Warsaw, Poland; 3 Department of Telecommunication, Informatics and Electronics, Bydgoszcz University of Science and Technology, al. Prof. Kaliskiego 7, 85-796 Bydgoszcz, Poland

**Keywords:** Optics and photonics, Optical materials and structures, Materials for optics

## Abstract

Mirrors are used in optical sensors and measurement setups. This creates a demand for mirrors made of new materials and having various properties tailored to specific applications. In this work, we propose silicon covered with a thin silicon nitride layer as a mirror for near-infrared measurements. SiN layer was deposited on a standard silicon wafer with a Low-Pressure Chemical Vapor Deposition furnace. Then, the created layer was investigated using ellipsometry and scanning electron microscope. Subsequently, the mirror was used as a reflecting surface in a Fabry–Perot fiber-optic interferometer. The mirror performance was investigated for wavelengths used in telecomunication (1310 nm and 1550 nm) and then compared with results obtained with the same measurement setup, with a silver mirror instead of silicon covered with SiN, as reference. Results showed that the proposed mirror can replace the silver one with satisfying results for investigated wavelengths.

## Introduction

Optical mirrors are one of the most frequently used optical components in optical sensors and measurement setups. Mirrors can be divided into two categories: metallic mirrors and dielectric mirrors. Metallic mirrors use a thin layer of metal as a reflecting surface^1^. Dielectric mirrors consist of multiple layers of material with high and low refractive indices^2^. High reflectivity is achieved by multiple Fresnel reflections from the layers. In optical systems and measurement setups, metallic mirrors are most commonly used; in very demanding applications these are silver mirrors in particular^3,4^. These mirrors are characterized by high reflectivity and a wide spectrum of reflected wavelengths^5^, but they have several drawbacks. The biggest problem during biological and medical measurements is silver chemical and biological reactivity^6,7^ when measured samples must be in direct contact with the mirror. A measured sample can react with mirror material thus changing its chemical composition or biological properties, which can influence measurement results. Another drawback of silver mirrors is their low mechanical damage threshold as they can be scratched very easily. Mirror installed in frequently used devices can quickly wear off, thus causing the need for frequent service and additional costs. In addition, silver mirrors, even with a protective layer, are vulnerable to corrosion^8–10^. Instead of standard metallic mirrors, silicon-based mirrors can be used, as they show good mechanical properties^11,12^. Research on replacing silver as a reflecting surface has been going on research group. As part of them, materials with optical properties similar to silver, and at the same time devoid of its disadvantages, have already been proposed. These include diamonds doped with boron^13^ or nitrogen^14^. Their parametric properties are close to silver. They are also biocompatible. Their disadvantage is the difficulty of their manufacture and the potentially high cost of production. A very interesting alternative is SiC^15^. Due to its mechanical and thermal resistance^16^, it is used as a reflecting layer in space applications^17^. Another interesting material that can be used to fabricate a mirror is carbon nanotubes^18^ in epoxy matrix. Due to material’s light weight and low modulus of elasticity, it can be used as active telescope mirror^19^.

In this work, we propose a silicon coated with a layer of silicon nitride (SiN) as a mirror for fiber-optic sensors. Silicon nitride is characterized by low chemical reactivity^20–22^, biocompatibility^23^ and in addition high hardness and superior wear resistance^20,22,24^. Thus, it can eliminate the aforementioned problems with standard mirrors, enabling trouble-free application in optical measurements of biological samples. We implemented silicon covered with silicon nitride as a mirror in a Fabry–Perot fiber-optic interferometer working in a reflective mode, and compared results with measurements performed with a silver mirror. In our study, we examine mirrors in a range of wavelengths used in fiber-optic telecommunication. Fiber-optic telecommunication has three most commonly used bands called telecommunication windows: the first is around 850 nm, the second around 1300 nm, and the third around 1550 nm^25^. First window is currently not as frequently used as second and third, due to the higher signal absorption than in second and third^25^. Using telecommunication range of wavelengths assures several advantages. The biggest one is wide access to devices and parts, such as light sources, optical fibers, couplers, etc., that can be easily adapted to sensors working with this range of wavelengths, at reasonable prices. Moreover, sensors able to operate at these wavelengths can be coupled with existing telecommunication systems.

## Materials and methods

### Materials

#### Preparation and characterization of SiN

The silicon wafer (4″, 1–10 Ohm cm, $\langle {100} \rangle$) was cleaned and prepared using following procedure:

- SC1 (Standard Clean 1), DIW:NH_4_OH:H_2_O_2_ 5:1:1 at 80 °C (353 K) for 10 min;
- SC2 (Standard Clean 2) DIW:HCl:H_2_O_2_ 6:1:1 at 80 °C (353 K) for 10 min;
- HF (hydrofluoric acid) 5% in DIW (deionized water) for 5 min.

**Figure 1:**
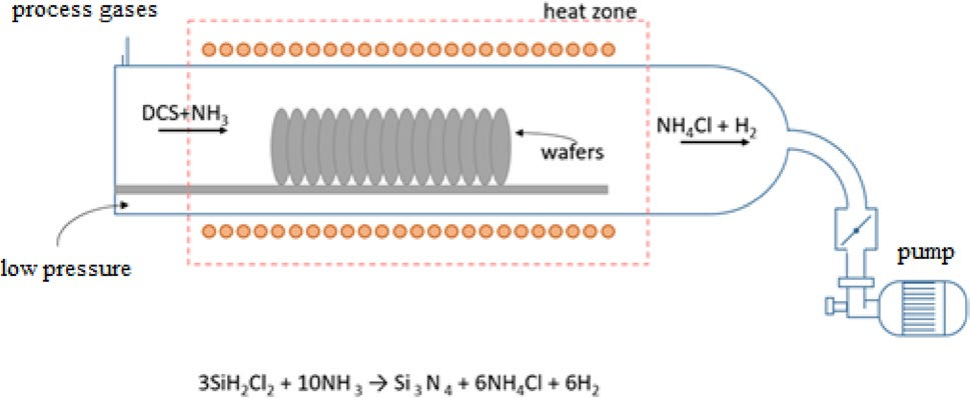
Scheme of LPCVD furnace used for the SiN deposition process.

Afterwards, the silicon wafer was processed in a horizontal LPCVD (Low-Pressure Chemical Vapor Deposition, SYSTEM ASM 2803, THERMCO SYSTEM, UK) furnace (Fig. 1) to deposit silicon nitride (SiN) layer^16^.

The pressure in the furnace tube was set at 200 mTorr and the temperature during the process was 770 °C (1043 K). Ammonia (NH_3_) and dichlorosilane (H_2_SiCl_2_) in a ratio of 1:5 were used as process gases.

#### SiN layer characterization

The first method of layer characterization was ellipsometry (UVISEL 2-NIR, HORIBA, France). The four-inch wafer was mapped at 225 points. The results are shown in Table 1.

**Table 1:** Results of thickness measurements and the value of the refractive index n parameter for the deposited SiN layer.

	Average	Maximum	Minimum	Standard deviation Ñ	Uniformity
Thickness (nm)	492.019	517.085	475.440	12.295	0.042
Reflactive index n (at 633.0 nm)	2.141	2.141	2.141	0.000	0.000
Extinction coefficient k (at 633.0 nm)	0.002	0.002	0.002	0.000	0.000
Fitting error Χ^2^	2.517	3.844	1.834	0.578	0.354

**Figure 2:**
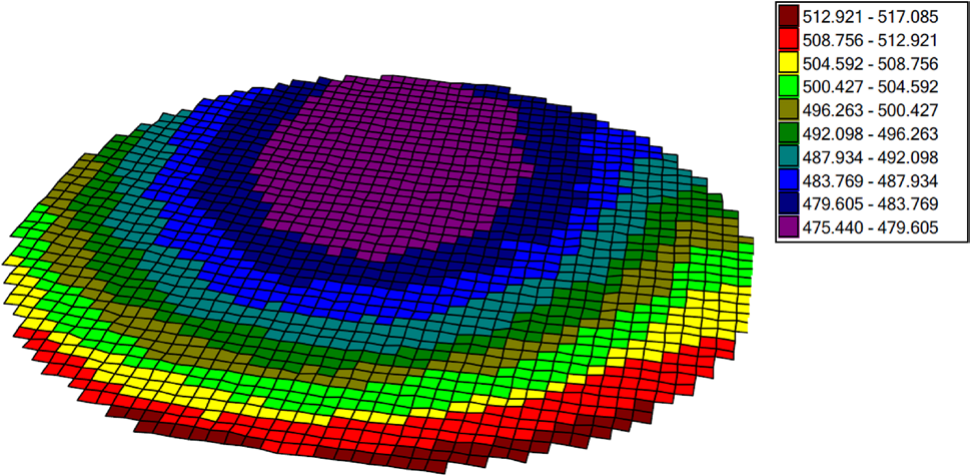
Map of the SiN layer thickness after the deposition process using the LPCVD method (red dotted line shows cleave direction).

A thickness map was prepared based on the ellipsometric measurements and it is shown in Fig. 2. The thickness variations resulted from wafer orientation in the oven during silicon nitride deposition, but the overall uniformity was high and in the expected range.

The layer thickness pattern is information about the uniformity of the layer. The more homogeneous the obtained layer is, the better, as it provides more reproducible results regardless of the measuring point on its surface. This is a great advantage when using a given layer in a sensor. The tested layer has differences of up to several dozen nanometers, which is much smaller than the wavelengths of 1330 nm and 1550 nm. For this reason, these differences will not affect the measurement, as they are too small to be observed with the measurement setup was use.

**Figure 3:**
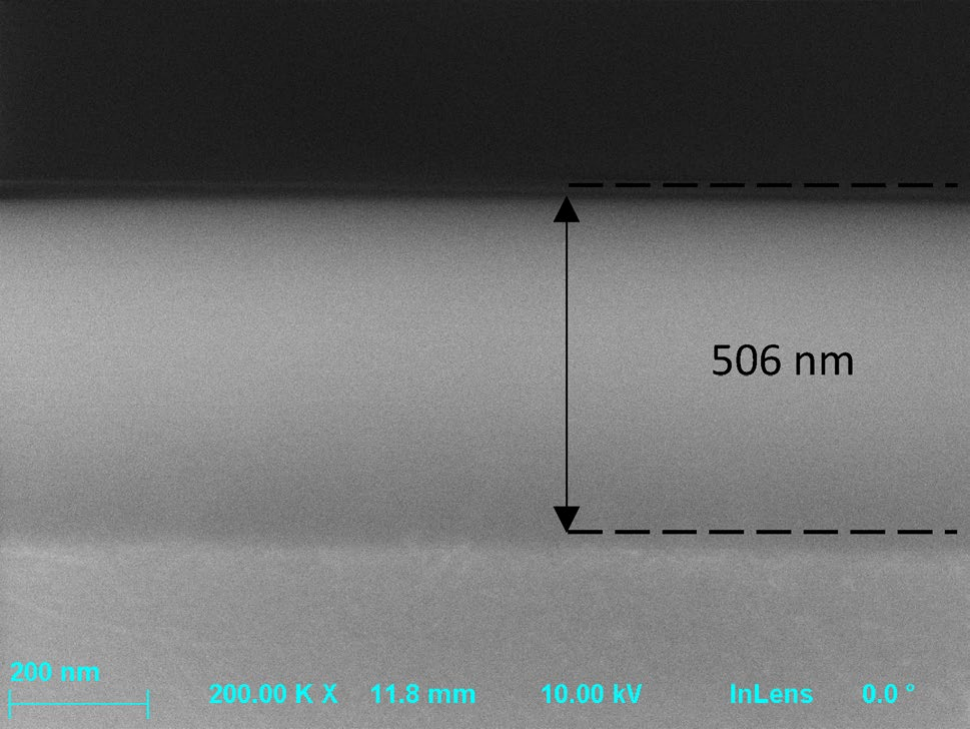
SEM image of central point of the Si substrate with the SiN layer deposited.

SiN layer thickness was confirmed with SEM (Scanning Electron Microscope, AURIGA 60, ZEISS, Germany). The wafer was cleaved into two halves (Fig. 2) and its thickness was measured by SEM (Fig. 3) in the central point of the cleave (center of the wafer).

### Methods

#### Measurement set-up

**Figure 4:**
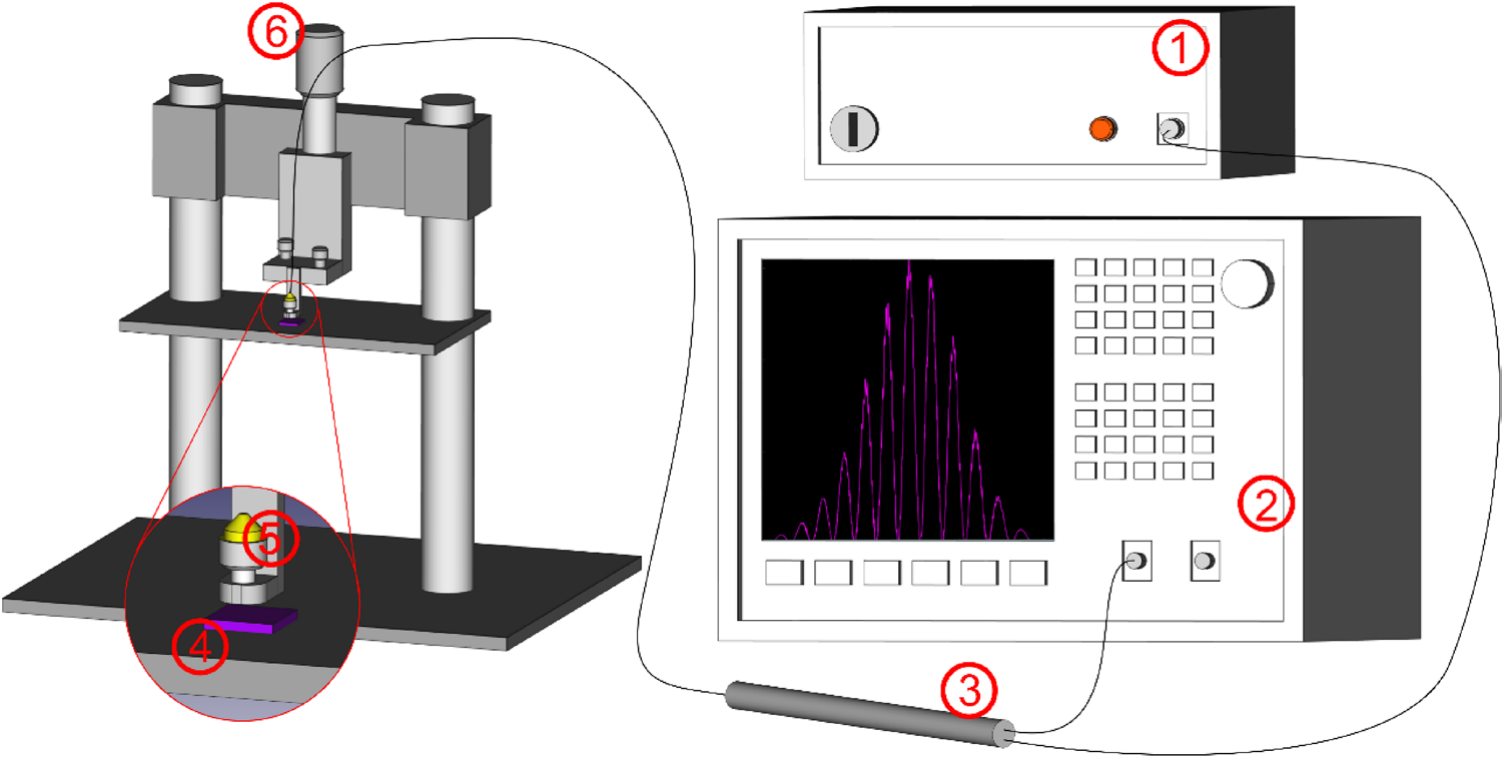
Measurement setup, 1—light source, 2—optical spectrum analyser, 3—fiber coupler, 4—investigated SiN layer, 5—fiber end-face, 6—micrometric screw.

Interferometric measurements were conducted using a fiber-optic Fabry–Perot interferometer working in a reflective mode. A simplified schematic of the measurement setup is presented in Fig. 4.

The light source and the spectrum analyser were connected with the sensing interferometer using telecommunication single-mode fibers coupled by a coupler (Lightel, WA, USA) with a 1 × 2 power split (50/50 symmetrical system). The first coupler arm was connected to the light source. During measurements, we used 2 different light sources. The first light source was a 1550 nm Fibre Labs Inc SLD-1550-13 and the second one was a 1310 nm Fibre Labs Inc SLD-1310-18. The second coupler arm was connected to the spectrum analyser (Ando model AQ 6319). The end of the third coupler arm was creating an interferometer cavity using its fiber end-face and Si wafer covered with SiN layer as reflecting surfaces. The fiber end-face was placed in parallel to the surface of SiN layer with a small gap between them creating the resonating cavity. Its width was modified using a micrometric screw that allowed adjusting width of the cavity with a 10 μm accuracy. Silver was used under the same conditions for the reference measurements.

## Results

### Results of interferometric measurements

The purpose of the measurements was to examine changes in the spectral characteristics, thus all of them were standardized. Two major factors were responsible for the shape of these characteristics: spectra of the light sources were responsible for the envelope of the characteristics while the appearance of the peaks was caused by light interference in the Fabry–Perot resonator. Peaks are observed for wavelengths corresponding to the resonating frequency of the resonance cavity. Position and distance between the maxima are a function of the cavity width.

The optical spectrum of the Fabry–Perot interferometer SF(v) is the result of the convolution of the source signal S(v) and the transmission of the interferometer T(v). For interferometers, they can be represented by the Formula (1)^26^

1 $$ SF(v) = S(v) \times T(v) = S(v) \times \left[ {1 + \cos \frac{4\pi nl}{\lambda }} \right], $$

where: S(v) is the optical spectrum of the source signal, n is the refractive index of the medium inside the cavity, l is the physical light path length, λ is the wavelength.

The shape of the spectral characteristics thus depends on the characteristics of the source used and the optical path length. Based on formula (1), an interferometric model was created. Its detailed description is presented in the literature^27^. The model was used to calculate the theoretical values of the optical parameters of the Fabry–Perot interferometer using Ag and SiN mirrors.

**Figure 5:**
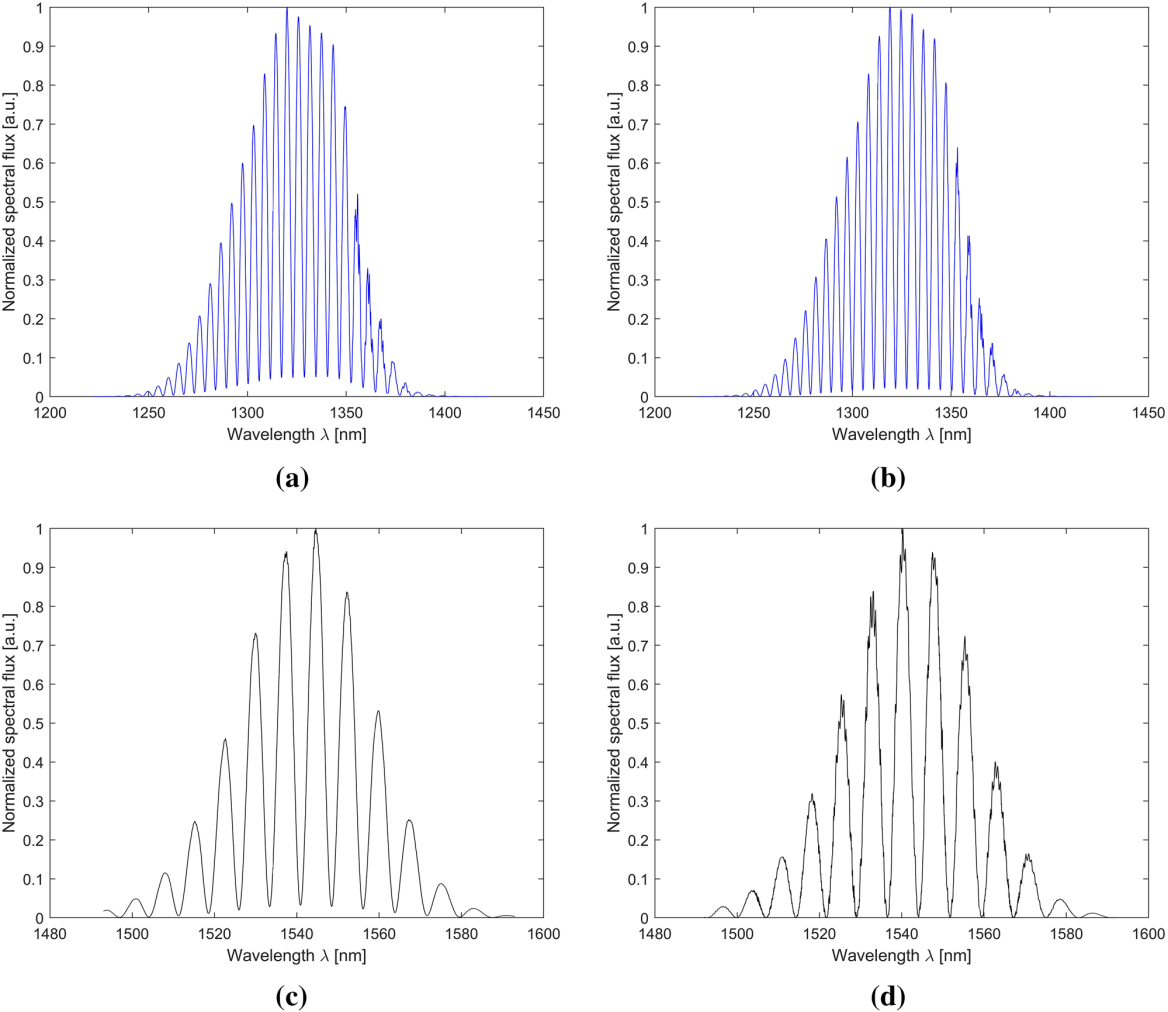
Selected optical characteristics measured for the cavity length of 150 μm on (**a**) the silver mirror for the wavelength of 1310 nm; (**b**) the SiN thin film for the wavelength of 1310 nm; (**c**) the silver mirror for the wavelength of 1550 nm; (**d**) the SiN thin film for the wavelength of 1550 nm.

Representative optical characteristics are shown in Fig. 5. It shows two optical spectra for each source: a reference measurement for the silver mirror, and a measurement for the SiN layer. All the presented measurements of the characteristics were made for the same width of the resonance cavity equal to 150 μm. This made it possible to directly compare the obtained results.

**Figure 6:**
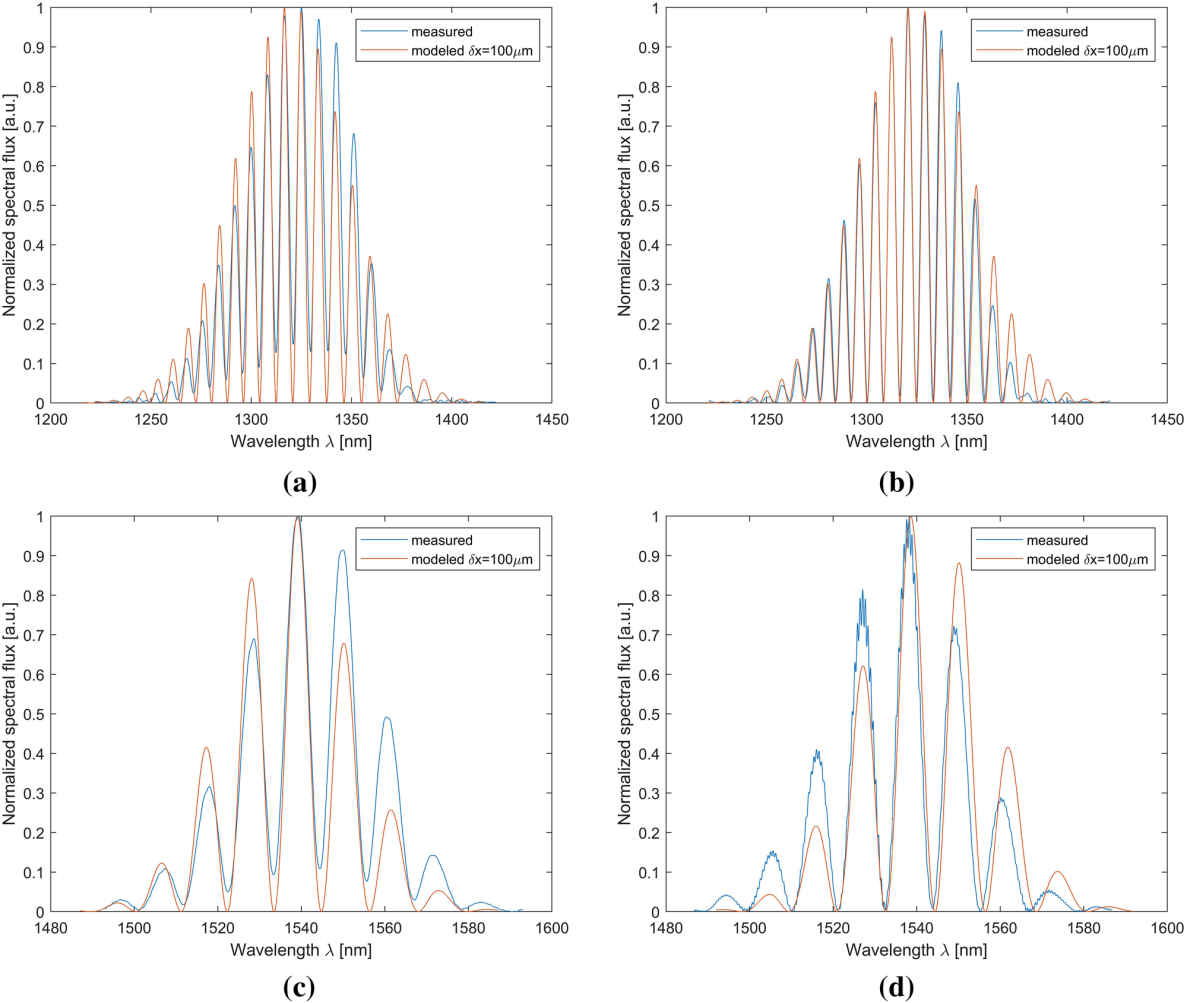
Comparison of the theoretical optical spectra with the measurement results (**a**) for a silver mirror at a wavelength of 1310 nm; (**b**) for an SiN thin film at a wavelength of 1310 nm; (**c**) for a silver mirror at a wavelength of 1550 nm; (**d**) for a SiN thin film at a wavelength of 1550 nm. The cavity length was set to 100 µm.

The comparison of the selected spectra shows similiarity. For a wavelength of 1310 nm, the optical spectra are almost identical, differing only in the level of absorption. It is bigger for the measurement carried out with the silver mirror. This shows that the tested layer can provide better optical parameters than silver. For a wavelength of 1550 nm, the optical spectra have identical modulation with slight phase shift.The measured optical spectra are consistent with the modeled ones in terms of distance between adjacent peaks as shown in Fig. 6.

The measured optical spectra fit their theoretical models. The shape of the obtained characteristics is matching, as well as the position of maxima in spectra. The measured characteristics are differing from the model in terms of intensity values, which can be caused by the slight displacement of the measurement head and losses due to absorption appearing in a non-ideal measurement environment. Appearing mismatches on the wavelength axis result from the adopted source model. The real source was approximated with ideal Gaussian characteristics, assuming perfect symmetry of the distribution which resulted in negligible mismatches on the wavelength axis.

The optical spectra in the function of the cavity length of the interferometer were measured. The range of the cavity length was changed from 20 to 200 µm with an increment of 10 µm. Measurements were made using both light sources (1310 nm and 1550 nm). In the investigation, two mirrors were examined: a thin film of SiN and a silver mirror as reference. All measurements were repeated 10 times to verify their repeatability. The optical properties were analyzed based on the spectral separation between the maxima. These values include information about the modulation of the signal.

## Discussion

The best way to observe the difference in measured spectra is to calculate the distance between corresponding peaks in one characteristic and compare the results with each other. Observation of the position of the specific peak can be difficult due to light source fluctuations over time. Calculation of distance between peaks makes measurement results invulnerable to changes in the light source. The distance between the peaks was calculated as the difference between the wavelength of the occurrence of the central peak and the wavelength of its nearest neighbouring peak. The distance to the right and left of the central peak was checked for each spectrum. Boxplot charts were used to show the statistical analysis of the measurement data. They show the mean value, the lower and upper quartile of data. Both quartiles form a rectangle with the mean value in the middle. They use whiskers that indicate the minimum and maximum values. In some cases, indicators of outliers of the entire data group appear marked with crosses. This chart type is insensitive to outliers and keeps the information about the data distribution. Its advantage is the small number of measurements required to obtain reliable data^28,29^.

**Figure 7:**
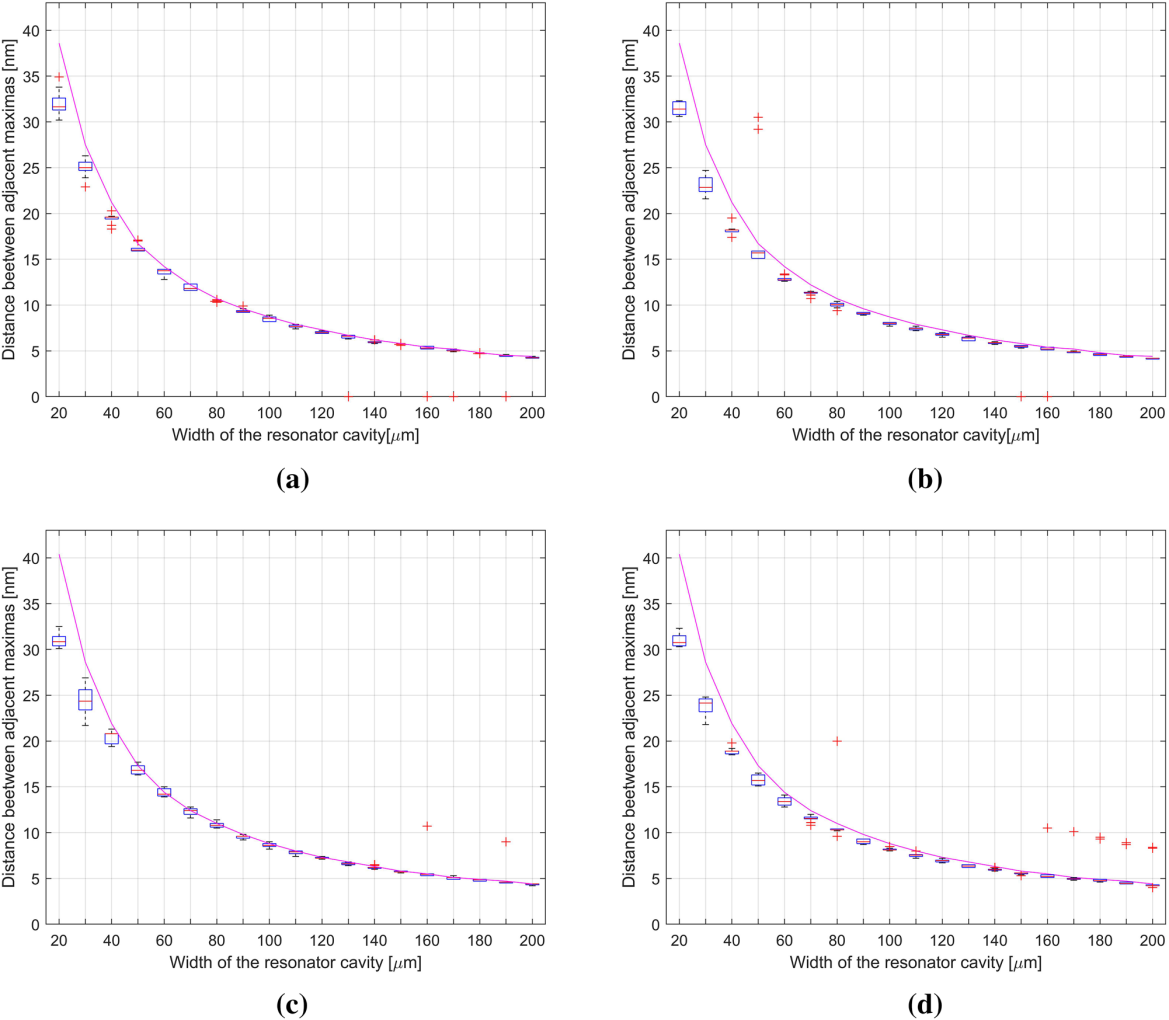
Distance between adjacent maxima in the signal using a 1310 nm source: (**a**) measured between the central and left peaks for silver mirror; (**b**) measured between the central and left peaks for SiN thin film; (**c**) measured between the central and right peaks for silver mirror; (**d**) measured between the central and right peaks for SiN thin film (where the magenta line is a theoretical model of data, red horizontal line is the mean value, red crosses are outliers).

Figure 7 shows boxplot charts for reference measurements and measurements of SiN at 1310 nm. They present changes of distance between the peaks in the range of tested cavity widths. For each material two charts are shown: one presenting the distance on the left side of the central peak, and the other showing the distance on the right side of the central peak. A theoretical values of the changes in the distance between the adjacent peaks were plotted in Fig. 7.

The Fig. 7a shows the left-hand scattering of values for silver does not exceed 3 nm, and it decreases as the width of the cavity increases. The best repeatability of the measurement was obtained for the cavity widths of 80, 150, and 180 μm. For these widths, the measurement series difference did not exceed 0.5 nm. Measurements for the right side are most reproducible in the range from 90 to 200 μm. The differences between series are around 0.5 nm. For the cavity length values below 90 μm, the repeatability is smaller. This can be due to the limited accuracy of setting the cavity length with the micrometer screw. The difference between the data in the series is 0.5 to 1 nm.

For SiN on the left side, we observe the spread of data at the level of 0.7 nm or less for the width range from 60 to 200 μm. The best repeatability was obtained for 140 μm and 200 μm. Changes between series of measurements do not exceed 0.1 nm. For the right side, the largest data spread is 2 nm. From 70 μm, the dispersion is smaller than 0.8 nm. The best measurement repeatability is for the widths of 140, 150, 170, and 200 μm. For these widths, the measurement series differed by only 0.2 nm.

From Table 2 it can be seen that the greatest differences between the model and the mean of the data occur for the width from 20 μm to about 40 μm for silver and 70 μm for SiN Silver parameters from the width of 50–200 μm are similar to the model. Their difference does not exceed 0.5 nm. Such compliance with the SiN model is achieved in the width range of about 100–200 μm.

**Table 2:** Comparison between the theoretical model and the average data of 1310 nm.

Value of difference between the model and the average		≥ 1 nm	1 nm ≤ 0.5	< 0.5	The greatest compliance
**For silver**					
Civity of width (μm)	Left side of spectrum	20–40 μm	–	40–200 μm	190 μm
	Right side of spectrum	20–40 μm	–	40–200 μm	180 μm
**For SiN**					
Cavity of width (μm)	Left side of spectrum	20–70 μm	70–120 μm	120–200 μm	190 μm
	Right side of spectrum	20–50 μm	60–100 μm	100–200 μm	140 μm

Both materials show similar properties. The measurements for silver were closest to the model from the width of 40 μm and for SiN from about 100 μm. Measurement repeatability at the level of 0.5 nm difference between series was obtained for silver in the range of 90–200 μm μm, and for SiN from 70 to 200 μm.

**Figure 8:**
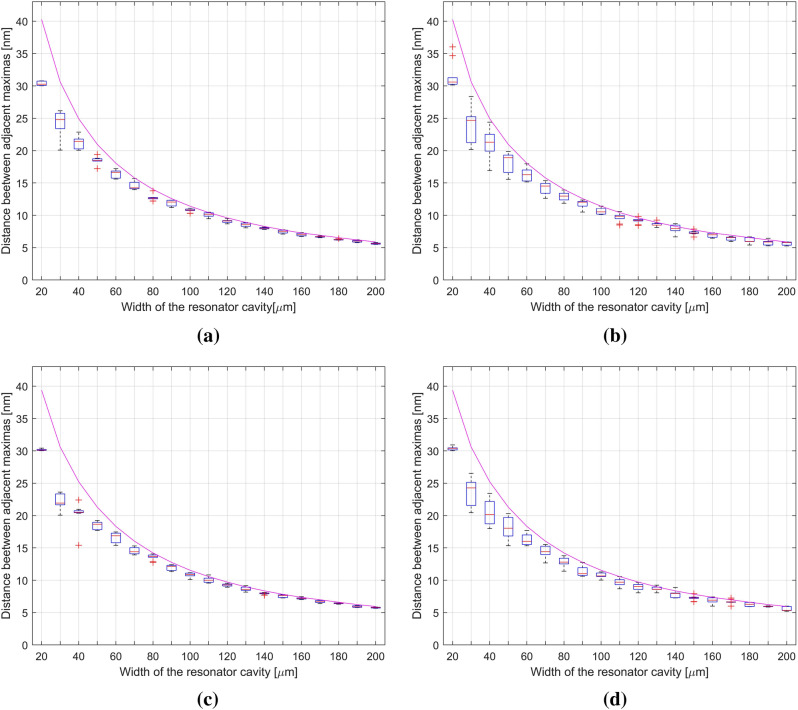
Distance between adjacent maxima in the signal using a 1550 nm source: (**a**) measured between the central and left peaks for silver mirror; (**b**) measured between the central and left peaks for SiN thin film; (**c**) measured between the central and right peaks for silver mirror; (**d**) measured between the central and right peaks for SiN thin film (where the magenta line is a theoretical model of data, red horizontal line is the mean value, red crosses are outliers).

Figure 8 shows the plots of the distance between the peaks dependent on the width of the resonant cavity. Measurements were made using a 1550 nm source, theoretical lines showing modeled values of changes in the distance between the peaks are shown in each plot. For SiN on the left, the smallest distributions were observed for widths from 110 to 200 μm. The differences between the series of measurements must not exceed 1 nm. The best measurement repeatability was obtained for the width of 130 μm. The difference between the highest and the lowest value in the series was about 0.9 nm. On the right, repeatability with a data spread of 1 nm was observed for a width of 110 to 200 µm. The most reproducible measurements were obtained for the width of 190 μm. The difference between the maximum and minimum measurement values was 0.2 nm. The silver data distribution on the left shows a data spread of less than 1 nm for widths from 90 µm. The best reproducibility was observed for the width of 140 μm. The difference between the highest and the lowest measured value is 0.3 nm. On the right, repeatability with a spread of less than 1 nm was observed for widths from 80 to 200 µm. The best repeatability was obtained for the widths of 180 and 200 μm. The data distribution for these widths did not exceed 0.2 nm. Differences in the distribution of data for SiN and silver were observed. The data spread is greater for SiN in the range from 20 to 110 μm. Most likely, it is the fault of an under-regulated micromechanical screw. For widths above 110 μm, both materials have a data spread lower than 1 nm. The differences between the SiN distribution and the silver distribution in this range differ by tenths of nm.

From Table 3 it can be seen that the greatest differences between the model and the mean of the data were observed for the range of 20–70 μm for silver and for the range from 20 μm to about 90 μm for SiN. The silver closest to the model was in the range from 70 to 200, and SiN in the range from 140 to 200 μm.

**Table 3:** Comparison between the theoretical model and the average data of 1550 nm.

Value of difference between the model and the average data		≥ 1 nm	1 nm ≤ 0.5	< 0.5	The greatest compliance
**For silver**					
Cavity of width (μm)	Left side of spectrum	20–70 μm	–	70–200 μm	160 μm
	Right side of spectrum	20–70 μm	70–110 μm	110–200 μm	180 μm
**For SiN**					
Cavity of width (μm)	Left side of spectrum	20–80 μm	80–140 μm	140–200 μm	200 μm
	Right side of spectrum	20–100 μm	100–130 μm	130–200 μm	130 μm

For both materials at a wavelength of 1550 nm, similar values of the tested parameters were observed. Fitting to the model at a difference of 1 nm between the mean and the model was observed for SiN from 80 μm, and for silver from 70 μm. Measurement repeatability with 1 nm data spread for SiN was observed from 110 μm, and for silver from 90 μm. For both materials, the best data spread was 0.2 nm.

One of the most important metrological parameters in interferometry is visibility. It was calculated for individual characteristics following the formula^30^:

$$ V = \frac{{I_{max} - I_{min} }}{{I_{max} + I_{min} }} $$

where: V—visibility, *I*_*max*_—highest signal intensity, *I*_*min*_—lowest signal intensity.

Table 4 presents the calculaed visibility values. The calculations were performed for both investigated wavelengths. The results obtained during the measurements of the SiN thin film. The visibility values have been shown for two cavity widths—80 μm and 150 μm. This selection allows the analysis of the visibility behavior in the range of the tested cavity widths.

**Table 4:** Comparison of the visibility values for the tested SiN material and silver for the second and third wavelengths of the telecommunications window.

Materials	Wavelength 1310 nm		Wavelength 1550 nm	
	Cavity width (μm)	Visibility (a.u)	Cavity width (μm)	Visibility (a.u)
Silver mirror	80	0.7446	80	0.7815
	150	0.9593	150	0.9687
SiN thin film	80	0.9644	80	0.9430
	150	0.9801	150	0.9974

The tested SiN thin film has higher visibility values than silver. This is independent of the wavelength used. There is a significant difference between the values for a cavity width of 80 µm. The visibility for silver was about 0.2 lower than for SiN thin film. This means that the SiN thin film can be used for measurements on narrower cavity widths than silver.

## Conclusions

The performed measurements and their analysis showed that the SiN thin film can replace silver as the reflecting layer in the interferometer. The produced tested film can be considered homogeneous as the changes in its thickness were insignificant and did not change the refractive index. During optical measurements, the SiN layer behaved similarly to silver in terms of signal modulation. The performed statistical tests proved that the tested layer provides repeatable measurements for a resonant cavity with a width in the range of 20–200 μm. Comparing the tested material in terms of visibility with silver and doped diamond films described in the literature, it turned out that it shows very good optical properties. It can work in smaller cavities than silver, which is significant in biological measurements. This allows smaller samples to be tested. SiN thin film also has better performance for larger cavity widths than silver. Summing up, the tested SiN layer can replace silver as it assures better optical parameters in a greater distance measurement range. Moreover, it introduces biocompatibility and greater wear resistance.

## Data Availability

The data used in this investiagtion can be requested from the corresponding authors.

## References

[1] Feller, A. *et al.* in Reflectivity, polarization properties, and durability of metallic mirror coatings for the European Solar Telescope (eds Navarro, R., Cunningham, C. R. *et al*.) 84503U (2012). 10.1117/12.927080.

[2] Ștefanov T (2020). Thin film metallic glass broad-spectrum mirror coatings for space telescope applications. J. Non-Cryst. Solids X.

[3] Boccas M, Vucina T, Araya C, Vera E, Ahhee C (2006). Protected-silver coatings for the 8-m Gemini telescope mirrors. Thin Solid Films.

[4] Song D-Y, Sprague RW, Macleod HA, Jacobson MR (1985). Progress in the development of a durable silver-based high-reflectance coating for astronomical telescopes. Appl. Opt..

[5] Born M (1999). Principles of Optics: Electromagnetic Theory of Propagation, Interference and Diffraction of Light.

[6] Lin J-J, Lin W-C, Li S-D, Lin C-Y, Hsu S (2013). Evaluation of the antibacterial activity and biocompatibility for silver nanoparticles immobilized on nano silicate platelets. ACS Appl. Mater. Interfaces.

[7] da Rocha Camargo E (2021). Plasma-assisted silver deposition on titanium surface: Biocompatibility and bactericidal effect. Mater. Res..

[8] Schwinde S, Schürmann M, Jobst PJ, Kaiser N, Tünnermann A (2015). Description of particle induced damage on protected silver coatings. Appl. Opt..

[9] Folgner KA (2020). Development and growth of corrosion features on protected silver mirrors during accelerated environmental exposure. Appl. Opt..

[10] Chu C-T, Fuqua PD, Barrie JD (2006). Corrosion characterization of durable silver coatings by electrochemical impedance spectroscopy and accelerated environmental testing. Appl. Opt..

[11] Wang X, Wang C, Shen X, Sun F (2017). Potential material for fabricating optical mirrors: Polished diamond coated silicon carbide. Appl. Opt..

[12] Steinlechner J (2018). Silicon-based optical mirror coatings for ultrahigh precision metrology and sensing. Phys. Rev. Lett..

[13] Majchrowicz D, Kosowska M, Struk P, Jędrzejewska-Szczerska M (2017). Tailoring the optical parameters of optical fiber interferometer with dedicated boron-doped nanocrystalline diamond thin film. Phys. Status Solidi A.

[14] Kosowska M (2021). Incorporation of nitrogen in diamond films—A new way of tuning parameters for optical passive elements. Diamond Relat. Mater..

[15] Tsuno, K. *et al.* New-technology silicon carbide (NT-SiC): Demonstration of new material for large lightweight optical mirror. in (eds Komar, G. J., Wang, J. *et al*.) 138 (2005). 10.1117/12.580660.

[16] Abrego Serrano PA (2020). Spherical mirror and surface patterning on silicon carbide (SiC) by material removal rate enhancement using CO_2_ laser assisted polishing. Int. J. Precis. Eng. Manuf..

[17] Ding G, He R, Zhang K, Zhou N, Xu H (2020). Stereolithography 3D printing of SiC ceramic with potential for lightweight optical mirror. Ceram. Int..

[18] Saleh T (2012). Transforming carbon nanotube forest from darkest absorber to reflective mirror. Appl. Phys. Lett..

[19] Chen PC, Rabin D (2014). Carbon nanotube optical mirrors. J. Astron. Telesc. Instrum. Syst.

[20] Lattemann M, Nold E, Ulrich S, Leiste H, Holleck H (2003). Investigation and characterisation of silicon nitride and silicon carbide thin films. Surf. Coat. Technol..

[21] Riley FL (2004). Silicon nitride and related materials. J. Am. Ceram. Soc..

[22] Tatarko P, Lojanová Š, Dusza J, Šajgalík P (2010). Influence of various rare-earth oxide additives on microstructure and mechanical properties of silicon nitride based nanocomposites. Mater. Sci. Eng. A.

[23] Neumann A (2004). Comparative investigation of the biocompatibility of various silicon nitride ceramic qualities in vitro. J. Mater. Sci. Mater. Med..

[24] Liang G (2021). Mechanical and dielectric properties of functionalized boron nitride nanosheets/silicon nitride composites. Ceram. Int..

[25] Phillips RL (1993). Book Rvw: Fiber optic communications. By Joseph C. Palais. Opt. Eng..

[26] Egorov SA, Mamaev AN, Polyantsev AS (1995). Spectral signal processing in intrinsic interferometric sensors based on birefringent polarization-maintaining optical fibers. J. Lightwave Technol..

[27] Pawłowska S (2021). Computer support of analysis of optical spectra measurements. Eng. Proc..

[28] Krzywinski M, Altman N (2014). Visualizing samples with box plots. Nat. Methods.

[29] Thirumalai, C., Vignesh, M. & Balaji, R. Data analysis using box and whisker plot for lung cancer. in *2017 Innovations in Power and Advanced Computing Technologies *(*i-PACT*) 1–6 (IEEE, 2017). 10.1109/IPACT.2017.8245071.

[30] Ferrari AC, Robertson J (2001). Resonant Raman spectroscopy of disordered, amorphous, and diamondlike carbon. Phys. Rev. B.

